# Thymoquinone Inhibits Bone Metastasis of Breast Cancer Cells Through Abrogation of the CXCR4 Signaling Axis

**DOI:** 10.3389/fphar.2018.01294

**Published:** 2018-12-04

**Authors:** Muthu K. Shanmugam, Kwang Seok Ahn, Annie Hsu, Chern Chiuh Woo, Yi Yuan, Kwong Huat Benny Tan, Arunachalam Chinnathambi, Tahani Awad Alahmadi, Sulaiman Ali Alharbi, Angele Pei Fern Koh, Frank Arfuso, Ruby Yun-Ju Huang, Lina H. K. Lim, Gautam Sethi, Alan Prem Kumar

**Affiliations:** ^1^Department of Pharmacology, Yong Loo Lin School of Medicine, National University of Singapore, Singapore, Singapore; ^2^College of Korean Medicine, Kyung Hee University, Seoul, South Korea; ^3^Cancer Science Institute of Singapore, National University of Singapore, Singapore, Singapore; ^4^Department of Botany and Microbiology, College of Science, King Saud University, Riyadh, Saudi Arabia; ^5^Department of Pediatrics, College of Medicine, King Khalid University Hospital, King Saud University Medical City, Riyadh, Saudi Arabia; ^6^Stem Cell and Cancer Biology Laboratory, School of Pharmacy and Biomedical Sciences, Curtin Health Innovation Research Institute, Curtin University, Perth, WA, Australia; ^7^Department of Obstetrics and Gynaecology, National University Hospital, Singapore, Singapore; ^8^Department of Anatomy, Yong Loo Lin School of Medicine, National University of Singapore, Singapore, Singapore; ^9^Department of Physiology, Yong Loo Lin School of Medicine, National University of Singapore, Singapore, Singapore; ^10^NUS Immunology Program, Life Sciences Institute, Centre for Life Sciences, National University of Singapore, Singapore, Singapore; ^11^Medical Sciences Cluster, Yong Loo Lin School of Medicine, National University of Singapore, Singapore, Singapore; ^12^Faculty of Health Sciences, Curtin Medical School, Curtin University, Perth, WA, Australia; ^13^National University Cancer Institute, National University Health System, Singapore, Singapore

**Keywords:** breast cancer, metastasis, thymoquinone, chemokine receptor 4, preclinical “*in vivo*” study

## Abstract

Overexpression of chemokine receptor type 4 (CXCR4) has been found to be associated with increased cell proliferation, metastasis and also act as an indicator of poor prognosis in patients with breast cancer. Therefore, new agents that can abrogate CXCR4 expression have potential against breast cancer metastasis. In this study, we examined the potential effect of thymoquinone (TQ), derived from the seeds of *Nigella sativa*, on the expression and regulation of CXCR4 in breast cancer cells. TQ was found to inhibit the expression of CXCR4 in MDA-MB-231 triple negative breast cancer (TNBC) cells in a dose- and time-dependent manner. It was noted that suppression of CXCR4 by TQ was possibly transcriptionally regulated, as treatment with this drug caused down-regulation of nuclear factor kappa-light-chain-enhancer of activated B cells (NF-κB) activation and suppression of NF-κB binding to the CXCR4 promoter. Pretreatment with a proteasome inhibitor and/or lysosomal stabilization did not affect TQ induced suppression of CXCR4. Down-regulation of CXCR4 was further correlated with the inhibition of CXCL12-mediated migration and invasion of MDA-MB-231 cells. Interestingly, it was observed that the deletion of p65 could reverse the observed anti-invasive/anti-migratory effects of TQ in breast cancer cells. TQ also dose-dependently inhibited MDA-MB-231 tumor growth and tumor vascularity in a chick chorioallantoic membrane assay model. We also observed TQ (2 and 4 mg/kg) treatment significantly suppressed multiple lung, brain, and bone metastases in a dose-dependent manner in a metastasis breast cancer mouse model. Interestingly, H&E and immunohistochemical analysis of bone isolated from TQ treated mice indicated a reduction in number of osteolytic lesions and the expression of metastatic biomarkers. In conclusion, the results indicate that TQ primarily exerts its anti-metastatic effects by down-regulation of NF-κB regulated CXCR4 expression and thus has potential for the treatment of breast cancer.

## Introduction

Breast cancer is the second most common cancer that afflicts women, with an estimated 1.67 million women diagnosed with breast cancer in 2012 (Bray et al., 2012; Torre et al., 2015). Breast cancer ranked fifth in cancer-associated deaths among all cancers globally in 2012 (Bray et al., 2012; Torre et al., 2015). While incredible development has been made for the treatment of breast cancers, the treatment of triple negative breast cancer (TNBC) is quite a challenge owing to its destructive features and limited treatment options (Jia et al., 2016; Wang et al., 2018). Often, chemotherapy for breast cancer is ridden with side effects and drug resistance, leading to failure in therapy. Compelling evidence suggests that if breast cancer metastasis to distant site organs can be prevented, then it is an indicator for good prognostic outcome (Alvarez et al., 2010; Perez and Spano, 2012). Interactions between chemokines and their cognate receptors play important roles in tumor metastasis. The interaction between chemokine receptor type 4 (CXCR4) and its cognate ligand stromal-derived factor-1 (SDF1 or CXCL12) plays a crucial role in the regulation of migration and metastasis in a variety of solid tumors including breast cancer (Balkwill, 2004a,b). Moreover, SDF1 has been found to be frequently over-expressed in lymph nodes, lung, liver, and bone marrow (Muller et al., 2001; Keeley et al., 2010). Hence, in the present study, we investigated the effect of thymoquinone on the SDF1/CXCR4 signaling axis.

Numerous novel bioactive compounds have been reported in several parts of medicinal plants that play an essential role in the prevention and treatment of chronic inflammation driven cancers (Shanmugam et al., 2011a, 2012, 2015, 2016; Sethi et al., 2012; Yang et al., 2013; Tang et al., 2014; Bishayee and Sethi, 2016; Hasanpourghadi et al., 2017). Thymoquinone (TQ) is one of the volatile bioactive constituents of *Nigella sativa* seed oil. Various studies have reported the anti-inflammatory, anti-oxidant, and anti-cancer properties of TQ both *in vitro* and *in vivo* (Shanmugam et al., 2018). TQ has been found to inhibit breast, ovarian, pancreatic, colon adenocarcinoma (Worthen et al., 1998; Shoieb et al., 2003; Gali-Muhtasib et al., 2004; Woo et al., 2012, 2013), human osteosarcoma (Roepke et al., 2007), lung carcinoma (Kaseb et al., 2007), myeloblastic leukemia (El-Mahdy et al., 2005), and multiple myeloma (Li et al., 2010; Siveen et al., 2014b) by modulation of diverse molecular targets that are involved in cell proliferation, survival, invasion, metastasis, and angiogenesis. *In vivo* TQ has been reported to inhibit the growth of tumors (Salem, 2005; Gali-Muhtasib et al., 2006; Woo et al., 2013). Moreover, TQ has been found to down-regulate inducible nitric oxide synthase and cyclooxygenase-2 (COX-2) (El-Mahmoudy et al., 2002; El Mezayen et al., 2006).

The master transcription factor nuclear factor kappa-light-chain-enhancer of activated B cells (NF-κB) plays a pivotal role in the development and progression of inflammation-driven diseases including cancer (Dey et al., 2008; Sethi et al., 2008b, 2012; Sethi and Tergaonkar, 2009; Shanmugam et al., 2013; Li et al., 2015; Liu et al., 2018; Puar et al., 2018). In human chronic myeloid leukemia cells (KBM-5), TQ was reported to abrogate NF-κB activation and augment cellular apoptosis (Sethi et al., 2008a). Several other studies have shown that TQ can also down-regulate protein kinase B and extracellular receptor kinase signaling pathways (Yi et al., 2008). Woo et al., 2011 reported that TQ can exert a strong anti-proliferative effects in TNBC cells by activating peroxisome proliferator-activated receptor gamma (PPARγ) (Woo et al., 2011). TQ administered intraperitoneally, has been found to be well tolerated up to 22.5 mg/kg in male rats and 15 mg/kg in female rats; whereas for TQ administered orally, the dose was as high as 250 mg/kg in both male and female rats (Abukhader, 2012).

Our prior published data has already indicated that TQ can exert anti-cancer effects on MCF7 breast cancer cells through activation of the PPARγ signaling cascade (Woo et al., 2011). In a recent study TQ was shown to suppresses the proliferation, migration, and invasion of metastatic MDA-MB-321 breast cancer cells by inhibiting the p38 mitogen-activated protein kinase pathway *in vitro* and *in vivo* (Woo et al., 2013). Therefore, we postulated that TQ may modulate the expression of CXCR4 and inhibit tumor metastasis *in vivo*. Our results indicate that TQ can down-regulate CXCR4 expression and CXCL12-induced migration and invasion in breast cancer cell lines through inhibition of NF-κB activation. Furthermore, we tried to investigate the effect of TQ on experimental metastasis that involved the injection of breast cancer cells directly into the systemic circulation (Hwang et al., 2015).

Breast cancer has a greater tendency to spread to the bone marrow of the femora, tibiae, and the mandibular bones. Therefore, intracardiac injection method has been employed to develop osteolytic bone metastasis model (van der Horst and van der Pluijm, 2012; Kumar and Manjunatha, 2013; Lee et al., 2014). On the basis of the crucial role of SDF1/CXCR4 in breast cancer metastasis to femora, tibiae, and mandibles, we systemically injected via an intracardiac route MDA-MB-231-luc^+^ cells to test the ability of TQ to modulate CXCR4 expression and thereby inhibit metastasis to the bones and other organs.

## Materials and Methods

### Cell Lines

Human breast cancer MCF7, MDA-MB-231, and BT-549 were purchased from ATCC (Manassas, VA, United States). The MDA-MB-231-luciferase expressing cell line was purchased from Cell Biolabs, United States. The cells were cultured in DMEM supplemented with 10% fetal bovine serum (FBS) and antibiotics penicillin/streptomycin.

### Materials

Thymoquinone (TQ), N-Acetyl-L-leucyl-L-leucyl-L-norleucinal (ALLN), chloroquine, formic acid, Tris, glycine, NaCl, sodium dodecyl sulfate (SDS), crystal violet, and bovine serum albumin were purchased from Sigma-Aldrich (St. Louis, MO, United States). A 50 mM solution of TQ was prepared with dimethyl sulfoxide (DMSO) and stored as small aliquots at -20°C. RPMI 1640, FBS, antibiotic-antimycotic mixture, and lipofectamine were obtained from Invitrogen (Carlsbad, CA, United States). Antibody against CXCR4 (polyclonal) was obtained both from Abcam (Cambridge, MA, United States) and Santa Cruz Biotechnology (Santa Cruz, CA, United States). CXCL12 was purchased from ProSpec-Tany TechnoGene, Ltd. (Rehovot, Israel). Antibody against β-actin (monoclonal), control siRNA and p65 siRNA was obtained from Santa Cruz Biotechnology (Santa Cruz, CA, United States). Ki-67 polyclonal antibody, Lamin B1, phospho-p65 (Ser 536), and p65 monoclonal antibodies were obtained from Cell Signaling Technology (Beverly, MA, United States). Goat anti-rabbit-horseradish peroxidase (HRP) conjugate and goat anti-mouse HRP were purchased from Sigma-Aldrich (St. Louis, MO, United States). D-Luciferin Firefly potassium salt was purchased from Gold Biotechnology, United States. Formic acid was purchased from Sigma-Aldrich (St. Louis, MO, United States). The NF-κB luciferase plasmid was obtained from Stratagene (La Jolla, CA, United States). The Bradford protein assay reagent was purchased from Bio-Rad Laboratories (Hercules, CA, United States). Isoflurane for anesthesia was purchased from National University of Singapore, Animal holding unit pharmacy (Singapore).

### Western Blotting

Vehicle or TQ-treated breast cancer cell lines and tumor tissues obtained from the mouse study were lysed in RIPA lysis buffer (Li et al., 2010). Nuclear extracts of TQ treated or control cells were prepared using TransAM nuclear extract kit according to the manufacturer’s instructions. Whole cell lysates was resolved on a 10% SDS polyacrylamide gel electrophoresis (SDS–PAGE) gel. The proteins were electro-transferred to a nitrocellulose membrane (Bio-Rad), blocked with Blocking One (Nacalai Tesque, Inc.), and probed with antibodies of interest overnight at 4°C. The blot was washed with *tris*-buffered saline with 0.1% Tween-20, exposed to HRP-conjugated secondary antibodies for 1 h, and finally examined by chemiluminescence using Western Bright Sirius HRP substrate (Advansta). Images were captured using a ChemiDoc XRS+ imaging system and analyzed using Image Lab^TM^ software (Bio-Rad, Hercules, CA, United States) as described previously (Chua et al., 2010). Densitometric analysis was done using ImageJ software.

### NF-κB Reporter Assay

MDA-MB-231 and BT-549 cells were plated in 6-well plates with 1 × 10^4^ cells per well in complete medium. The following morning, cells were transfected with an NF-κB reporter plasmid linked to a luciferase gene or with the dominant-negative IκBα (IκBα-DN) plasmid, co-transfected with a β-galactosidase plasmid (Promega, Madison, WI, United States). Transfections were done according to the manufacturer’s protocols using Lipofectamine (Invitrogen, United States). At 24 h post-transfection, cells were treated with TQ (50 μM) for indicated time points and then washed and lysed in luciferase lysis buffer (Promega). Luciferase activity was measured using a Tecan plate reader (Durham, NC, United States) by using a luciferase assay kit (Promega) and was normalized to β-galactosidase activity. All luciferase experiments were done in triplicate as previously described (Shanmugam et al., 2011b).

### NF-κB DNA-Binding Activity Assay

To determine NF-κB activation, nuclear extracts were prepared using a nuclear extraction kit (Active Motif, Carlsbad, CA, United States) according to the manufacturer’s instructions. NF-κB DNA-binding activity was analyzed using the TransAM NF-κB p65 transcription factor assay kit (Active Motif, Carlsbad, CA, United States), following the manufacturer’s instructions. The enzymatic product was measured at 450 nm with a microplate reader (Tecan Systems, San Jose, CA, United States) as previously described (Shanmugam et al., 2011b).

### Chromatin Immunoprecipitation and Quantitative RT-PCR

Human MDA-MB 231 and BT-549 TNBC cells were seeded in 15-cm culture dishes and treated with TQ (50 μM) for 0, 2, 4, 6, and 8 h. At the end of the treatment period the cells were fixed with 1% formaldehyde at room temperature for 10 min and neutralized with glycine. The cells were collected, resuspended in CHIP lysis buffer, and sonicated (Vibra-Cell,^TM^ Newtown, CT, United States). Samples were incubated with protein-G beads that had been pre-incubated with 4–10 μg of anti-NF-κB antibody (Cell Signaling Technology, MA, United States) or negative control IgG (Sigma-Aldrich Co., St. Louis, MO, United States). The next day, immunoprecipitants were washed by using washing buffer and reverse cross-linked at 65°C. The DNA was then purified by using a PCR purification kit purchased from MACHEREY-NAGEL (Duren, Germany). Finally, the purified DNA was subjected to quantitative RT-PCR and data analyzed.

### Scratch Wound Healing Assay

MDA-MB-231 and BT-549 cells were seeded in culture plate containing a culture-insert (Ibidi, Martinsried, Germany) until fully confluent. A cell-free gap of 500 μm was created after removing the culture-insert. After incubation with 50 μM TQ for 12 h, the medium was changed to medium with or without CXCL12. After migration for 8 or 24 h, the wound distance was observed and captured using bright field microscopy and the gap distance was measured using Photoshop software (Manu et al., 2011; Shanmugam et al., 2011b,c). MDA-MB-231 cells were transfected with 50 nmol/L of p65 or control siRNA. The cells were then subjected to wound healing assay either in the presence or absence of TQ (50 uM) for 8 h.

### Invasion Assay

An *in vitro* cell invasion assay was performed using a BioCoat Matrigel invasion assay system (BD Biosciences, San Jose, CA, United States), as described previously (Manu et al., 2013; Shanmugam et al., 2011b,c). MDA-MB-231 cells were transfected with 50 nmol/L of p65 or control siRNA. The cells were then subjected to invasion assay either in the presence or absence of TQ (50 uM) for 8 h.

### Determination of Tumor Growth Using a Chick Choriallantoic Membrane Assay

The chick chorioallantoic membrane (CAM) assay was modified from Sys et al. (2013). Briefly, fertilized chicken eggs (Bovans Goldline Brown) were purchased from Chew’s Agriculture Pte Ltd., Singapore and placed horizontally in a 37.5°C incubator with 70% humidity on embryonic day (ED)-0. On ED-3, a sharp weighted tool was used to poke a hole at the apex of the eggshell, and 3 mL of albumin was removed using a 5 mL syringe and 18G needle in order to drop the CAM. The sharp weighted tool was then used to poke a hole in the middle of the egg before using curved surgical scissors to cut a 1 cm^2^ hole. The eggs were screened and dead embryos were removed. The hole was then sealed with a 1624W Tegaderm semi-permeable membrane and the egg placed back into the incubator. On ED-7, MDA-MB-231 (0.65 × 10^6^) cells were mixed with matrigel. Fifty micro liter of the matrigel-cell mixture was placed on the CAM/egg. The hole was then re-sealed with the Tegaderm semi-permeable membrane. Twenty micro liter of DMSO or 25, 50, or 100 μM of TQ was added by pipetting onto autoclaved filter paper disks on ED-10 after the initial ultrasound scan. The tumor volume and tumor vascularity was determined at the 72 h time point in the control and TQ treated groups.

### Ultrasound Imaging

On embryonic day 10, and after 72 h incubation with or without TQ, the Tegaderm membrane was removed and Aquasonic gel was added onto cling wrap that had been carefully placed over the CAM tumors. Using a VisualSonics Vevo 2100 Imaging system, a 550D transducer connected to a 3D acquisition monitor was used to obtain ultrasound images of the tumors formed on the CAM. Parallel 2D sections obtained were further reconstructed to form 3D images of the tumors. Tumor volumes and percentage of vasculature were calculated using the Vevo Lab 1.7.0 program. On ED-13, after ultrasound imaging, the CAM tumors, along with chick liver (to check for metastasis) were carefully excised and washed in PBS, part of it was snap frozen in liquid nitrogen for molecular analysis while the other part was fixed in 10% formalin overnight at 4°C, before being embedded in paraffin. The paraffin blocks were then taken for future histopathological analysis.

### Intracardiac Experimental Metastasis Murine Model

All procedures involving animals were reviewed and approved by the NUS Institutional Animal Care and Use Committee. Seven week old NCr-Foxn1nu, female mice were purchased from InVivos Pte Ltd., Singapore. NCr-Foxn1nu female mice were intracardially implanted with 1 × 10^6^ MDA-MB-231-luc^+^ cell line. Female nude mice (age 7 weeks) were anesthetized with 3–5% isoflurane. On day 0, anesthetized animals were injected with 1 × 10^6^ MDA-MB-231-luc^+^ cells suspended in 100 μl sterile Dulbecco phosphate buffered saline (DPBS) into the left ventricle of the heart by non-surgical means (Contag et al., 2000). D-Luciferin potassium salt (Gold Biotechnology, St. Louis, MO, United States) at room temperature was dissolved in DPBS without calcium or magnesium to a final concentration of 15 mg/ml. The luciferin solution was sterilized using a 0.22 micron filter and then injected intraperitoneally at a concentration of 150 mg/kg body weight (b.w). The mice were placed in an imaging chamber and imaged both on the dorsal and ventral sides whilst under isoflurane anesthesia (3–5%) using an IVIS^TM^ Imaging System (Xenogen Corporation, United States), for 20 s–3 min as described previously (Rehemtulla et al., 2000; Jenkins et al., 2003a,b; Scatena et al., 2004).

A successful intracardiac injection was indicated on day 0 by images showing systemic bioluminescence distributed throughout the animal. Only mice indicating a satisfactory injection were included in the experimental groups. The mice were imaged once every week for tumor cell metastasis for a total of 4 weeks. Generally, two to three mice were imaged at a time. Imaging and quantification of signals were controlled by the acquisition and analysis software Living Image^®^ (Xenogen^®^ Corporation, United States). Tissues of interest were excised, placed into 24-well tissue culture plates with 300 μg/ml D-luciferin in DPBS and imaged for 1–2 min. Tissues were subsequently fixed in 10% formalin (Sigma, St. Louis, MO, United States) and prepared for standard histopathology evaluation.

### Hematoxylin and Eosin Staining

At the end of treatment, mice hind limbs and fore limbs were collected and fixed in 10% neutral buffered formalin solution (Sigma-Aldrich, St. Louis, MO, United States). The limbs’ muscle tissue was then removed to expose the bone. The bones were then decalcified by soaking in citric acid buffered formic acid (Sigma-Aldrich, St. Louis, MO, United States) for 96 h. The softened and decalcified bone was then processed and embedded into paraffin wax blocks. The bone samples were then sectioned and stained with hematoxylin and eosin solution (Merck, Germany). Images were taken using an Olympus BX51 microscope (magnification, 40×).

### Immunohistochemical (IHC) Analysis of Tumor Samples

Breast cancer tumor cells that had metastasized to the bone, lung, and brain from control and drug-treated groups were fixed with 10% phosphate buffered formalin, processed, and embedded in paraffin. The bone and tumor sections, 5 μM in thickness, were cut and deparaffinized in xylene, dehydrated in graded ethanols, and finally hydrated in water. Antigen retrieval was conducted by boiling the slide in 10 mM sodium citrate (pH 6.0) for 30 min. Sections were incubated overnight with anti-CXCR4 primary antibody (1:100 dilutions). IHC was conducted following the manufacturer’s instructions (Dako LSAB Kit) as described previously (Dai et al., 2015; Zhang et al., 2016). Images were taken using an Olympus BX51 microscope (magnification, 40×). Quantitative analysis of IHC images was performed by visual scores between the control and treated images. In this expression quantitation technique, each image was divided into four parts and each part was individually quantitated for the biomarker expression. A cell scored as positive refers simply to the presence of brown staining (peroxidase) in any part of the studied tissue. A negative cell scored refers to no staining or weak staining.

### Statistical Analysis

Student *t*-test was used to analyze the data. For *in vivo* studies, unpaired *t*-test was used for statistical comparisons between groups. *p* < 0.05 was considered statistically significant (GraphPad Prism 5.0; GraphPad Software, CA, United States).

## Results

**FIGURE 1:**
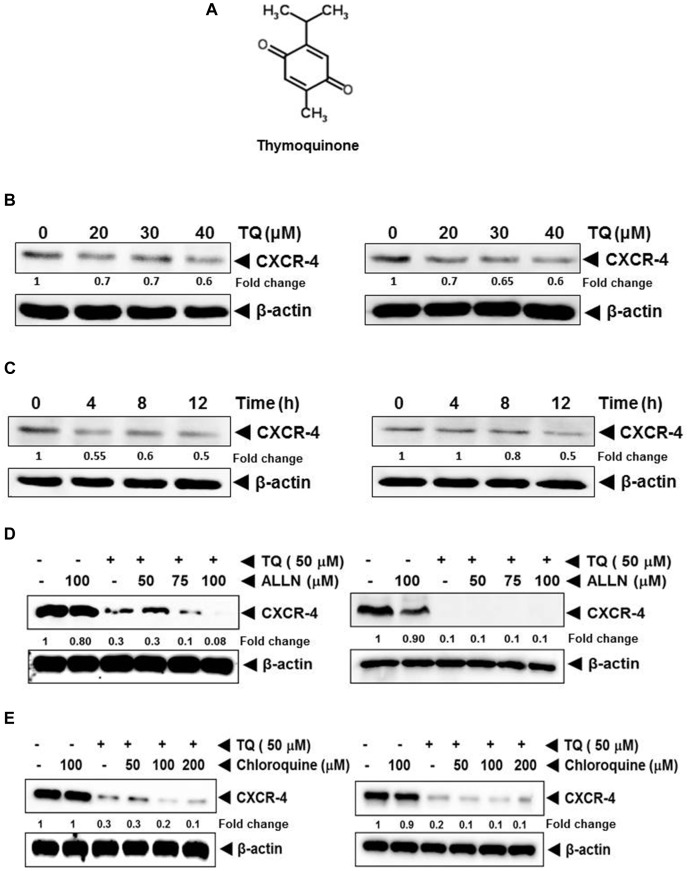
**(A)** The chemical structure of thymoquinone (TQ). **(B)** Western blot analysis of CXCR4 expression in breast cancer cells. TQ down-regulated the expression of CXCR4 in a dose-dependent manner. MCF7 (left panel) and MDA-MB-231 cells (right panel) were incubated with the indicated concentration of TQ for 12 h. Fifty micro gram of whole cell extracts of MCF7 and MDA-MB-231 cells were resolved on SDS–PAGE gel and probed with anti-CXCR4 antibody. The membrane were stripped and re-probed with anti-actin antibody to determine equal protein loading. **(C)** TQ suppressed CXCR4 expression in a time-dependent manner. MCF7 (left panel) and MDA-MB-231 (right panel) breast cancer cells were treated with 40 μM of TQ for the indicated time and Western blot analysis was performed as describe above. The blots were stripped and probed for actin as loading control. **(D)** TQ mediated suppression of CXCR4 was not through the proteasomal pathway. MDA-MB-231 cells (left panel) and BT-549 cells (right panel) were treated with the indicated concentration of ALLN for 1 h at 37°C followed by TQ (50 μM) for 12 h. Whole cell extracts were then subjected to Western blot analysis and probed for CXCR4. The same blots were then re-probed for actin to show equal protein loading. **(E)** TQ mediated suppression of CXCR4 was not through the lysosomal pathway. BT-549 cells were treated with the indicated concentration of chloroquine for 1 h at 37°C followed by TQ (50 μM) for 12 h. Whole cell extracts were then subjected to Western blot analysis and probed for CXCR4. The same blots were then reprobed for actin to show equal protein loading.

The present study was designed to investigate the effect of TQ (with structure shown in Figure 1A) on both CXCR4 expression and function in BT-549 and MDA-MB-231 TNBC cells and in a metastatic murine model.

### TQ Suppresses CXCR4 Protein Expression in Breast Cancer Cells

We first studied the effect of TQ on the expression of CXCR4 in MCF7 and MDA-MB-231 breast cancer cell lines. We observed that TQ could suppress the expression of CXCR4 in a dose- and time-dependent manner in MCF7 (Figures 1B,C, left panel) and MDA-MB-231 (Figures 1B,C, right panel), respectively.

### Down-Regulation of CXCR4 Expression by TQ Is Not Mediated Through Its Degradation

Previous studies have shown that CXCR4 undergoes ubiquitination at its lysine residue, followed by degradation (Kukreja et al., 2005; Shanmugam et al., 2011b); hence, we next examined the likelihood that TQ may augment the rate of CXCR4 degradation through the stimulation of proteasomes. MDA-MB-231 and BT-549 cells were pretreated with ALLN, a proteasome inhibitor, for 1 h before treating with TQ. As shown in Figure 1D, ALLN did not protect TQ-induced degradation of CXCR4, thereby indication that this is an improbable basis for the downregulation of CXCR4 expression by TQ. In addition we also studied the capability of chloroquine, a lysosomal inhibitor, to abrogate TQ-induced degradation of CXCR4, as CXCR4 has been shown to undergo ligand-dependent lysosomal degradation (Shanmugam et al., 2011b). Both the cell lines were pretreated with chloroquine for 1 h before being treated with TQ. Our results indicated that chloroquine at 200 μM only marginally prevented the degradation of CXCR4 (Figure 1E), thereby suggesting that this was possibly not the main pathway for suppression of expression of CXCR4.

### TQ Modulates Constitutive Activation of NF-κB in TNBC Cells

**FIGURE 2:**
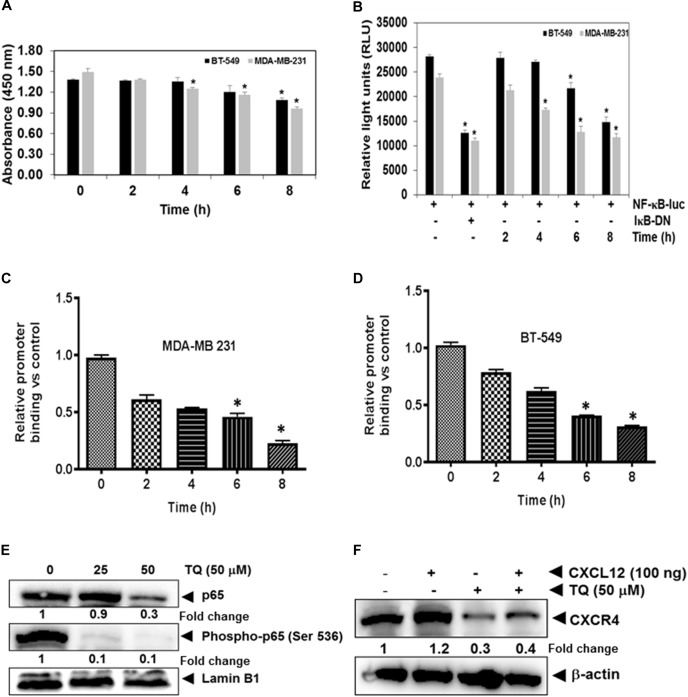
TQ modulates constitutive NF-κB activation in MDA-MB-231 and BT-549 cells. **(A)** Both breast cancer cell lines were incubated with 50 μM TQ for the indicated time points. The cells were then lysed in a hypotonic buffer and the nuclear extracts were prepared and assayed for NF-κB activation using TransAM p65 transcription factor assay kit. **(B)** Both MDA-MB-231 and BT-549 cells were transiently transfected with NF-κB luciferase plasmid and co-transfected with β-galactosidase plasmid for 6 h. The medium was changed and then treated with 50 μM TQ or inhibitory kappaB (IκB) plasmid for the indicated time points. The cells were then lysed in reporter lysis buffer (Promega Inc.) and assayed for luciferase activity as described in the Materials and Methods. Results are plotted as fold activity compared to vector only control. Bars indicate standard error. ^∗^Indicates *p*-value < 0.05. **(C,D)** TQ inhibits binding of NF-κB to the CXCR4 promoter. MDA-MB-231 cells **(C)** and BT-549 cells **(D)** were treated with 50 μM TQ for the indicated time points and the proteins were then cross-linked with DNA using formaldehyde and then processed for chromatin immunoprecipitation assay using anti-p65 antibody with CXCR4 primers as described in materials and methods. **(E)** Effect of TQ on p65 expression and phosphorylation in MDA-MB-231 cells. Nuclear extracts were prepared as described in Materials and Methods. MDA-MB-231 cells were treated with TQ at doses of 25 and 50 μM for 8 h and expression of various proteins was analyzed by western blot analysis. **(F)** TQ suppressed CXCL12 induced CXCR4 expression. MDA-MB-231 breast cancer cells were treated with 50 μM of TQ for 8 h, whole cell extracts was prepared and Western blot analysis was performed as describe in Materials and Methods. The blots were stripped and probed for actin as loading control.

The NF-κB pro-survival signaling pathway plays a critical role in breast cancer cell progression and metastasis (Helbig et al., 2003). The promoter region of the *CXCR4* gene also has several NF-κB binding sites (Shanmugam et al., 2011b). Therefore it is possible that TQ suppresses NF-κB activation and downregulate CXCR4 expression. We found that treatment with TQ suppressed NF-κB activation in a time-dependent manner (Figure 2A). This result suggests that TQ may down-regulate CXCR4 expression through inhibition of NF-κB activation. However, DNA binding alone is not always associated with NF-κB -dependent gene transcription, suggesting that added controlling steps are involved. Subsequent results also indicated that TQ inhibited NF-κB reporter activity in a time-dependent manner in TNBC cells (Figure 2B). As both phosphorylation and nuclear translocation are critical for the function of transcription factors, we next evaluated the effect of TQ on nuclear translocation of p65 and phospho-p65 expression by Western blot analysis. We found that TQ dose-dependently suppressed nuclear accumulation of p65 and phospho-p65 expression in the nucleus of MDA-MB-231 cells (Figure 2E).

### TQ Abrogates CXCL12-Induced Over-Expression of CXCR4

Chemokine receptor type 4 is one of the most important chemokine receptors in TNBC. High CXCR4 expressing tumor cells have a greater invasive and metastatic potential, and CXCR4^+^ cells migrate following a concentration gradient of CXCL12. Overexpression of this receptor has been observed in patients who exhibit advanced stages of cancer and metastasis and correlates with worse prognosis and decreased patient survival (Kato et al., 2003). We next investigated if indeed TQ can suppress CXCL12 induced overexpression of CXCR4 in MDA-MB-231 cells. Interestingly, we found that TQ suppressed CXCL12 induced over-expression of CXCR4 in MDA-MB-231 cells thereby indicating that positive feedback loop between CXCR4 and downstream signaling components may be mediated in an autocrine manner (Figure 2F).

### TQ Attenuates NF-κB Transcriptional Activity by Decreasing Its Binding to the CXCR4 Promoter

Whether the down-regulation of CXCR4 by TQ in TNBC cells was due to suppression of NF-κB activation *in vivo* was examined by a ChIP assay targeting NF-κB binding in the CXCR4 promoter. We found that TQ suppressed NF-κB binding to the CXCR4 promoter (Figures 2C,D) in both the TNBC cell lines, thereby indicating that TQ inhibits CXCR4 expression by suppressing NF-κB binding to the CXCR4 promoter. According to the quantitative RT-PCR result, compared to the untreated samples, TQ significantly decreased the binding of NF-κB to the CXCR4 promoter and this reduction was noted to occur in a time-dependent fashion (Figures 2C,D).

### TQ Inhibits CXCL12-Induced Cellular Invasion and Migration

**FIGURE 3:**
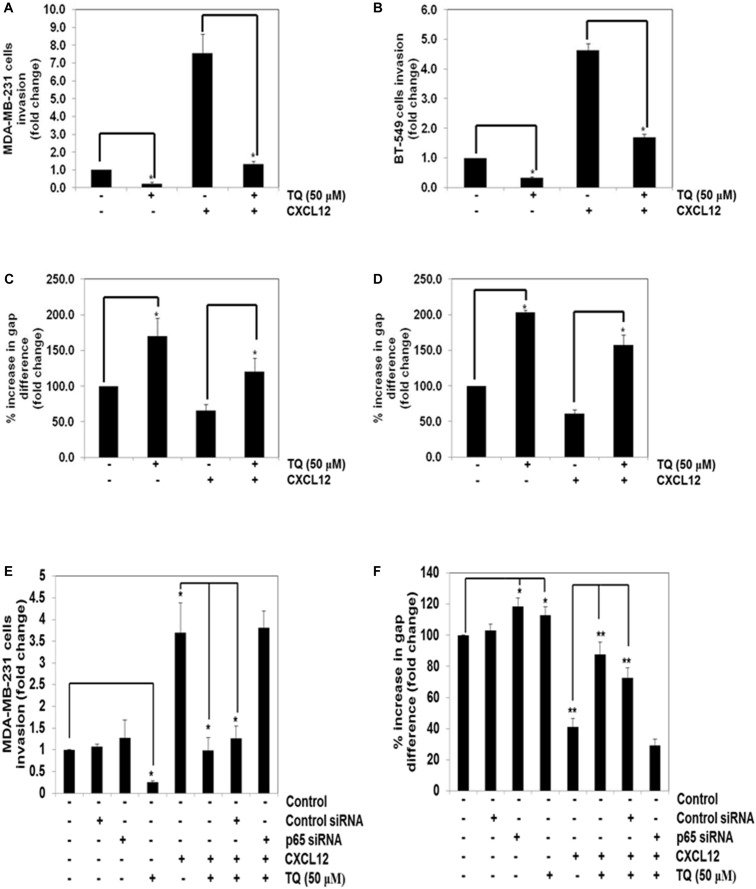
TQ abrogates invasion and migration of breast cancer cells. **(A,B)** Effect of TQ on invasion of MDA-MB-231 cells **(A)** and BT-549 cells **(B)**. The cells were seeded in the top side of the chamber and matrigel was coated as a layer on the bottom side of the chamber. The cells were pretreated with or without TQ (50 μM) for 12 h and the cells were then seeded in the top chamber and placed in a 24 well plate containing either the basal medium only or basal medium containing 100 ng/ml CXCL12 for 24 h. The invaded cells were then fixed and stained with 1% crystal violet solution. Columns represent the number of cells invaded. Bars indicate standard error. ^∗^Indicates *p*-value < 0.05. **(C,D)** Effect of TQ on migration of MDA-MB-231 and BT-549 cells. A scratch wound healing assay was performed to evaluate the inhibitory effect of TQ on breast cancer cell migration. A chamber containing 500 micron size cell free width was measured on day 0 and after incubation with 50 μM TQ for 12 h, the medium was changed to medium with or without CXCL12 (100 ng/ml). After migration for 24 h, the gap distance of the wound was measured at three different sites. Bars indicate standard error. ^∗^Indicates *p*-value < 0.05. **(E)** MDA-MB-231 cells were transfected with 50 nmol/L of p65 or control siRNA. The cells were then subjected to invasion assay either in the presence or absence of TQ (50 μM) for 8 h. **(F)** MDA-MB-231 cells were transfected with 50 nmol/L of p65 or control siRNA. The cells were then subjected to migration assay either in the presence or absence of TQ (50 μM) for 8 h. ^∗^Indicates *p*-value < 0.05; ^∗∗^Indicates *p*-value < 0.005.

We further elucidated the effect on TQ on CXCL12-induced cell invasion. Using an *in vitro* invasion assay, TQ treatment suppressed CXCL12-induced invasion in both the cell lines (Figures 3A,B). Furthermore, we performed addition experiment by using p65 siRNA to inhibit the NF-κB signaling pathway and to determine the anti-invasive potential of TQ on MDA-MB-231 cells. Interestingly, we found that upon inhibition of NF-κB, TQ was not able to significantly mitigate cellular invasion (Figure 3E). Whether the down-regulation of CXCR4 by TQ correlates with cell migration was also examined using an *in vitro* scratch wound healing assay. We found that both MDA-MB-231 and BT-549 cells migrated faster under the influence of CXCL12 and this effect was abolished on treatment with TQ (Figures 3C,D). Interestingly, we found that upon inhibition of NF-κB using p65 siRNA, TQ was not able to significantly attenuate cellular migration (Figure 3F).

### TQ Abolishes Tumor Growth and Vascular Volume in a CAM Assay Model

**FIGURE 4:**
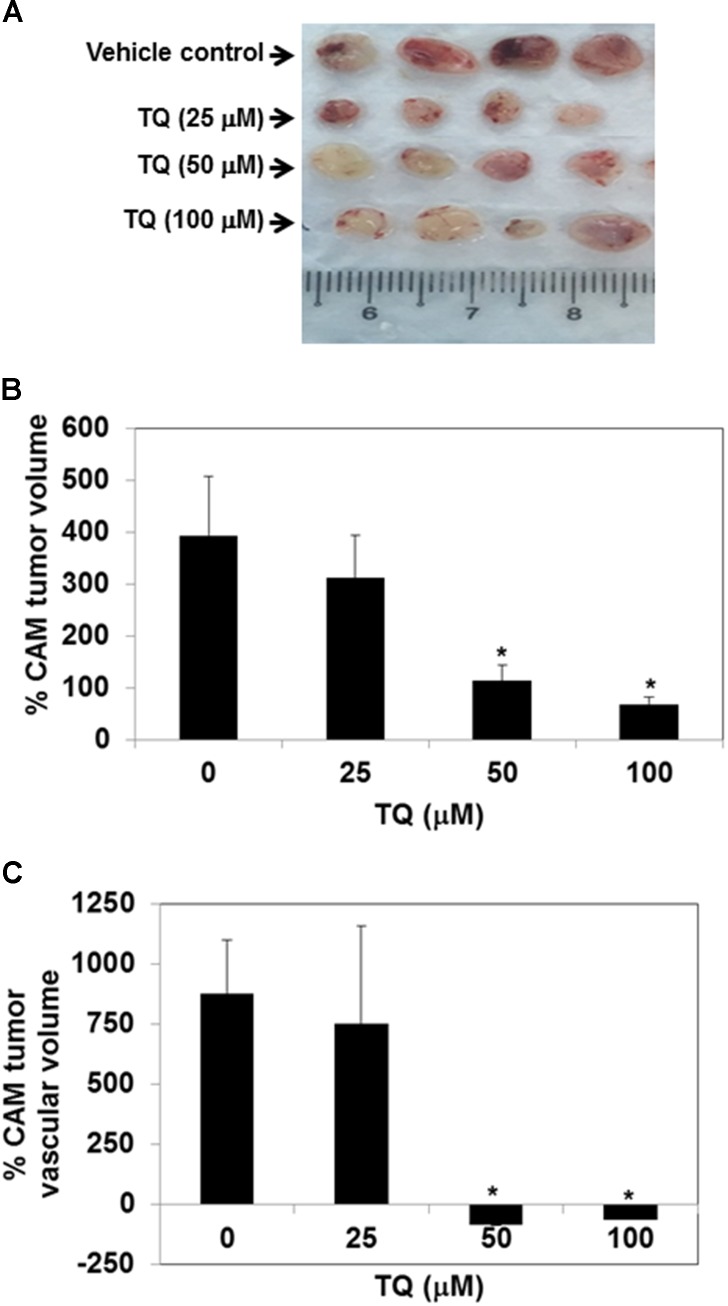
TQ abolishes the growth of MDA-MB-231 breast tumor cells inoculated onto chick chorioallantoic membrane and suppresses tumor vascular volume. **(A)** Representative images of MDA-MB-231 chorioallantoic membrane (CAM) tumors excised from the CAM. 0.65 × 10^6^ MDA-MB-231 cells were inoculated onto the CAM on embryonic day 7. The tumors were imaged by ultrasound on embryonic day 10. After the initial ultrasound, 20 μl of DMSO or 25, 50, or 100 μM TQ was added to the autoclaved filter paper disks kept on the CAM. A final ultrasound was taken on embryonic day 13 after 72 h treatment. **(B,C)** Effects of TQ on the growth of CAM tumor and tumor vascular volume. Tumor volumes were calculated using the Vevo Lab 1.7.0 program by tracing out the boundaries of the tumor and reconstruction of the 2D images. For each condition (*n* = 4) tumors were obtained and the average percentage change in tumor volumes **(B)** and percentage change in tumor vascular volumes were calculated **(C)**. DMSO-treated CAM tumors were significantly bigger and more vascularized than the TQ-treated tumors. Bars indicate standard error. ^∗^Indicates *p*-value < 0.05.

We evaluated the effect of TQ on the growth of tumors in a CAM assay model. We found that 3D ultrasound scanning of tumors indicated that TQ could inhibit the growth of tumors in a dose-dependent manner. TQ at doses of 50 and 100 μM significantly inhibited the growth of tumors compared to the vehicle control group (Figure 4B), and potently reduced the tumor vascular volume at doses of 50 and 100 μM, thereby indicating complete abrogation of tumor vasculature by TQ (Figure 4C). However, TQ at the lower dose of 25 μM had no effect on tumor growth and vascularity. Photographic images of CAM vehicle treated and TQ treated CAM tumors are shown in Figure 4A.

### TQ Inhibits Breast Cancer Bone Metastasis and Thinning of Bones

**FIGURE 5:**
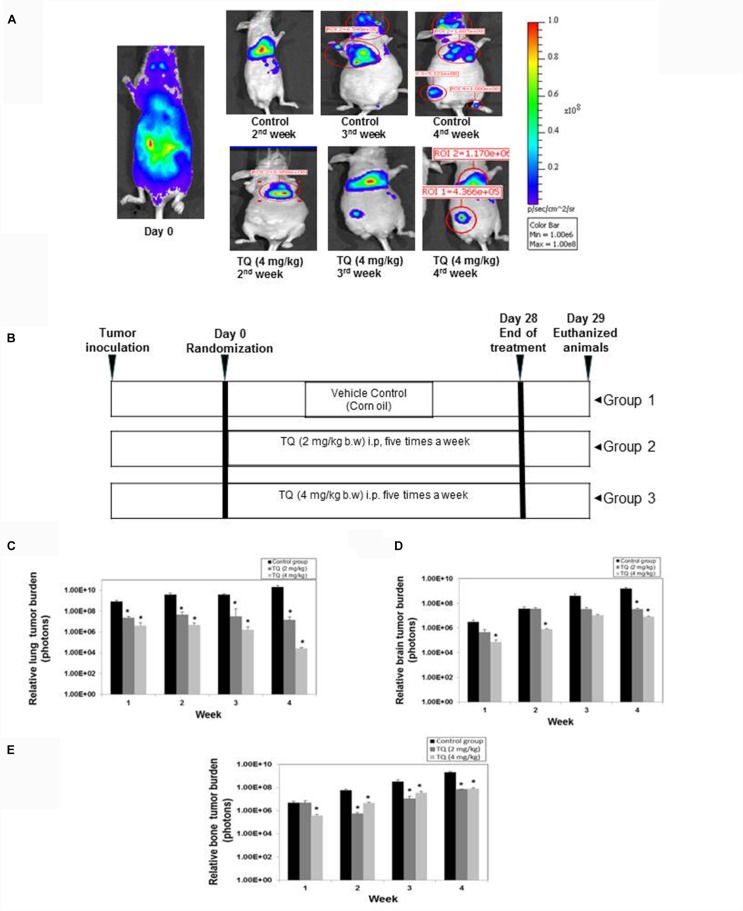
Anti-metastatic activity of TQ in a breast cancer metastatic model. **(A)** Representative images of bioluminescence imaging of mouse. **(B)** Schematic of dosing regimen. TQ at either 2 mg/kg or 4 mg/kg body weight was administered for 4 weeks. **(C)** Relative lung tumor metastasis in athymic nude mice bearing intracardially injected MDA-MB-231-Luc^+^ cells treated with either corn oil or with TQ (2 or 4 mg/kg b.w) for 4 weeks. **(D)** Relative brain tumor metastasis as determined by bioluminescence imaging. **(E)** Relative bone tumor metastasis as determined by bioluminescence imaging. Bars indicate standard error. ^∗^Indicates *p*-value < 0.05.

To investigate whether TQ can suppress bone metastasis, 8 weeks old nude mice were injected intracardiac with MDA-MB-231-luc^+^ expressing cells and then treated with TQ 2 mg or 4 mg/kg b.w. intraperitoneally for 4 weeks. The mice were divided into three groups and the schematic of the TQ dosing regimen is shown in Figure 5B. Bioluminescence images showed a decrease in number of colonies metastasized to distant site organs compared to vehicle control animals (Figure 5A). A significant dose-dependent, anti-metastatic effect was observed in the lung tissue (Figure 5C) and in the brain tissue (Figure 5D). High CXCR4 expressing MDA-MB-231 cells always tend to move to a region rich in CXCL12 such as bone marrow (Fernandis et al., 2004; Hung et al., 2014). By the third and fourth week we detected bone metastasis. At 4 weeks, metastatic activity was greater in control mice compared to TQ treated mice (Figure 5E).

**FIGURE 6:**
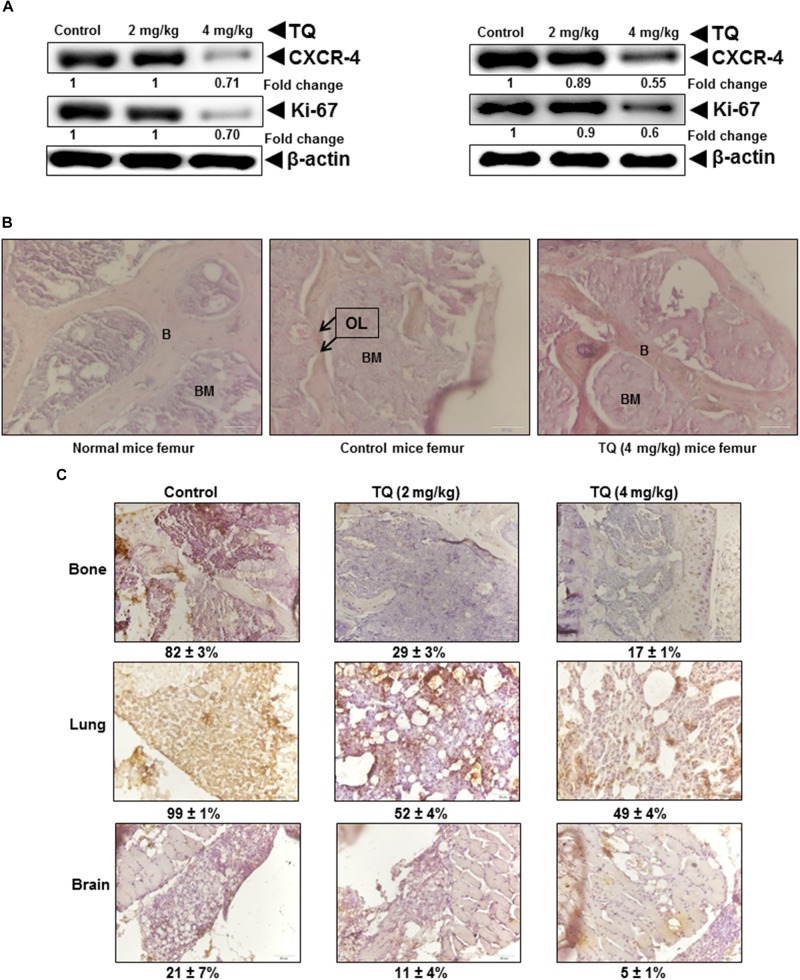
TQ abrogates the expression of metastatic genes in tumor tissue. **(A)** Metastatic lung (left panel) and brain (right panel) tumor tissue obtained from either vehicle control or TQ treated mice was lysed and subjected to Western blot analysis. Fifty micro gram of whole cell extracts were resolved in SDS–PAGE gel and probed for anti-CXCR4, anti-Ki67, and anti-β-actin. The membrane were stripped and re-probed with anti-actin antibody to determine equal protein loading. **(B)** Hematoxylin and eosin staining of metastatic femur bone obtained from normal mice, vehicle control mice, and TQ treated mice. B, trabecular bone; BM, bone marrow, black arrows indicate osteolytic lesion (OL) of trabecular bone. Images were taken using an Olympus BX51 microscope (magnification, 40×). **(C)** Immunohistochemical analysis of metastatic bone, lung, and brain tumor tissue. **(C)** Top panel, metastatic tissue obtained bone was stained for CXCR4 expression. A marked decrease in the expression of CXCR4 was observed in the femur of mice treated with TQ (4 mg/kg b.w.). A similar decrease in the expression of CXCR4 was observed in both lung [**(C)** middle panel] and brain tissue [**(C)** lower panel]. Images were taken using Olympus BX51 microscope (magnification, 40×).

Remarkably, it was found that TQ significantly suppressed the metastasis of breast cancer cells to the bone (Figure 5E), and also there was a decrease in the metastatic sites such as bone marrow of the femora, tibiae, and mandibles. We observed a decrease in metastatic osteolytic lesions using H&E staining, and a significant decrease in the bone colonization of breast cancer cells (Figure 6B). A similar anti-metastatic effect was also observed in the lung (Figure 5C) and brain tissues (Figure 5D). In addition, no significant decrease in the body weight was observed both in the vehicle treated control and TQ treatment group (data not shown). H&E examination of the bone sections revealed prevention of thinning of bones in the TQ treated group as compared to control mice (Figure 6B).

### TQ Reduces the Expression of NF-κB Regulated Gene Products in the Tumor Tissues

NF-κB regulates the expression of several genes that are involved in tumor growth, metastasis, and angiogenesis (Li et al., 2015). A dose-dependent decrease in the expression of NF-κB-regulated proteins (CXCR4 and Ki-67) in the lung and brain tissues was observed by immunoblot analysis (Figure 6A). As shown in Figure 6C, TQ treatment inhibited CXCR4 expression in bone (top panel), lung (middle panel), and brain tissues (bottom panel), that was assessed by IHC analysis. A marked decrease in the expression of CXCR4 was observed in the femur of mice, and a similar effect was observed in the lung and brain tissues treated with TQ (4 mg/kg b.w.).

## Discussion

The current study was designed to investigate the anti-metastatic effect of TQ, and its underlying molecular mechanisms of action in TNBC cell lines *in vitro* and in an intracardiac metastatic murine model. CXCR4 over-expression has been associated with tumor cell proliferation, invasion, and metastasis. TQ was found to inhibit the expression of CXCR4 in MDA-MB-231 and MCF7 breast cancer cell lines, irrespective of the cell type and HER2 status. We further observed that TQ inhibited constitutive NF-κB DNA binding and activation in both the TNBC cell lines and significantly suppressed NF-κB dependent luciferase expression. These results are in line with previous work showing that TQ can abrogate NF-κB activation pathway (Sethi et al., 2008a; Siveen et al., 2014b) in diverse cells. Interestingly, TQ mediated down-regulation of CXCR4 did not occur by proteolytic degradation of the receptor but rather by a decrease in the number of transcripts. In addition, inhibition of the CXCR4 receptor was associated with down-regulation of migration and invasion of TNBC cells upon stimulation with its ligand CXCL12. Numerous reports have previously suggested that CXCR4 is over-expressed in a variety of tumors including gastric, ovarian, pancreatic, melanoma, renal, cervical, colon, and hematological malignancies (Manu et al., 2011, 2013; Shanmugam et al., 2011b). However, there are no substantial studies elaborating upon the detailed mechanism(s) of CXCR4 over-expression in tumor cells (Kulbe et al., 2005).

Genetic mutations in *von Hippel Lindau* tumor suppressor gene (Staller et al., 2003), PAX3- and PAX7-FKHR gene fusion (Libura et al., 2002), angiogenic growth factors (Bachelder et al., 2002; Schioppa et al., 2003), deregulated NF-κB (Zlotnik, 2006), and pro-inflammatory cytokines (Kulbe et al., 2005) have all been implicated in CXCR4 over-expression. Recent evidence implicates that EGFR, c-erbB2 and its encoding gene *HER2/neu*, can also regulate CXCR4 expression at the post-transcriptional level in breast cancer cells (Benovic and Marchese, 2004; Arya et al., 2007). Moreover, studies have implicated CXCR4 over-expression to cancer metastasis and are an indicator of poor prognosis and overall patient survival (Xu et al., 2015). A high level of expression of CXCR4 was significantly associated with a greater risk of bone metastasis. Bone metastasis occurs significantly more often in CXCR4 positive patients (13.1%) as compared to CXCR4 negative patients (2.4%; *P* = 0.008) (Hung et al., 2014). In a recent article, Ashour et al. (2014, 2016) demonstrated that TQ can also inhibit NF-κB phosphorylation and its regulated gene products, such as interleukin-8 and chemokines, in a dose- and time-dependent manner in hepatocellular carcinoma cells. Therefore, CXCR4 appears to be a novel therapeutic target for the treatment of metastatic breast cancer. It has also been suggested that CXCR4 may be degraded by ligand-dependent lysosomal degradation (Fernandis et al., 2004) and by atrophin-interacting protein 4 mediated polyubiquitination and subsequent degradation (Bhandari et al., 2007).

CXCL12 is the only ligand for CXCR4 and acts as autocrine/paracrine growth factor for several cancers. Interestingly, we found that CXCL12 was able to increase the levels of CXCR4 in TNBC cells. In a recent study, it has been reported that the overexpression of both CXCR4 and CXCL12 mRNA in glioblastoma cancer stem cells (GBM-CSC) can control proliferation, invasion and angiogenesis. CXCL12 secretion from CXCR4 expressing cells supports the occurrence of autocrine/paracrine mechanism in GBM CSCs, in human meningioma and pituitary adenoma cells (Gatti et al., 2013). Furthermore, in another study by Borrello et al. (2005), it was reported that transforming oncogene *RET/PTC1* in human papillary thyroid carcinoma can induce the expression of CXCL12 and its receptor CXCR4, thus triggering autocrine proliferation of thyroid carcinoma cells (Borrello et al., 2005). Our results also demonstrate that TQ can substantially suppress CXCR4 expression in TNBC cells and this modulation was observed to occur at the transcriptional level and not mediated by either ligand-dependent lysosomal or proteasomal degradation. Because TQ was observed to modulate inhibit both NF-κB DNA binding and reporter gene expression, we further investigated the effects of TQ on CXCL12-induced invasion and migration in TNBC cell lines. We found that pre-incubation with TQ completely blocked CXCL12-induced migration and invasive potential of TNBC cells. Furthermore, TQ failed to abrogate inhibit invasion or migration of cells in p65 deleted cells.

We also investigated for the first time the potential effect of TQ on breast cancer cell bone metastasis in a breast cancer mouse model. CXCL12 signaling via CXCR4 has an important physiological role in hematopoietic stem cell homing to the bone marrow (Balkwill, 2004b; Xu et al., 2015). This signaling axis also plays a critical part in breast cancer cell metastasis by increasing chemotaxis to regions of high CXCL12 expression such as bone, lung, and brain (Arya et al., 2007). Therefore, down-regulation of CXCR4 in triple negative metastatic breast cancer cells could greatly decrease their metastatic potential. In our study we also determined the anti-cancer effects in a tumor-bearing chick embryo model. The CAM assay has been used extensively as a model for studying angiogenesis due to the accessibility and visibility of the developing blood vessels (Siveen et al., 2014a). However, recent studies have shown that tumor cells can grow readily on the CAM in an immunocompromised environment (Sys et al., 2013).

Tumor bearing chick embryos have been used to study anti-cancer effects of drugs (Abe et al., 2011; Busch et al., 2013). In our study, we implanted breast cancer cells onto the CAM and evaluated the effects of TQ on tumor growth and vascularity. We found that TQ dose-dependently inhibited tumor growth and potently reduced the tumor vascular volume. Furthermore, we found that intraperitoneal administration of TQ at doses of 2 or 4 mg/kg b.w for 4 weeks could inhibit the growth and metastasis of breast cancer tumors. TQ significantly suppressed lung, bone, and brain metastasis, as evidenced by a decrease in bioluminescence signals in these organs. It is of note that patients with terminal breast cancer exhibit severe bone destruction associated with osteolytic lesions (Mundy, 2002). We further evaluated the inhibitory effect of TQ on osteolytic bone metastasis in nude mice inoculated with MDA-MB-231-luc^+^ cells into the left cardiac ventricle. Bone metastasis was found to be significantly suppressed in the TQ treated group, and the number of osteolytic lesions in the mandibles, femora, and tibiae were markedly decreased, as indicated by bioluminescence imaging. Western blot analysis of tumors obtained from the lung and brain also showed a decrease in the expression of CXCR4 and Ki67. In fact, upon TQ treatment, there was a marked reduction in thinning of bones, as evidenced by H&E staining. In addition, IHC analysis of lung, brain, and bone tumors revealed inhibition of CXCR4 expression. Overall, TQ was well tolerated at the doses tested and was effective in suppressing the metastasis of TNBC cells. In conclusion, TQ inhibited NF-κB regulated CXCR4 expression, migration, as well as invasion of the TNBC cells, and markedly suppressed bone metastasis by suppressing NF-κB activation cascade. Further studies are warranted as TQ as its clinically relevant doses are yet to be determined.

## Author Contributions

GS, APK, KHBT, LL, RH, KSA, AC, TA, MS, and SA conceived the project. GS, APK, KHBT, LL, RH, and KSA designed the experiments. MS, AH, CW, YY, and APK carried out the experiments and analysis the data. MS, GS, AKo, APK, KSA, and FA wrote and corrected the paper.

## Conflict of Interest Statement

The authors declare that the research was conducted in the absence of any commercial or financial relationships that could be construed as a potential conflict of interest.

## References

[B1] Abe C. Uto Y. Nakae T. Shinmoto Y. Sano K. Nakata H. (2011). Evaluation of the *in vivo* radiosensitizing activity of etanidazole using tumor-bearing chick embryo. *J. Radiat. Res.* 52 208–214. 10.1269/jrr.10122 21436611

[B2] Abukhader M. M. (2012). The effect of route of administration in thymoquinone toxicity in male and female rats. *Indian J. Pharm. Sci.* 74 195–200. 10.4103/0250-474X.106060 23440704 PMC3574528

[B3] Alvarez R. H. Valero V. Hortobagyi G. N. (2010). Emerging targeted therapies for breast cancer. *J. Clin. Oncol.* 28 3366–3379. 10.1200/JCO.2009.25.4011 20530283

[B4] Arya M. Ahmed H. Silhi N. Williamson M. Patel H. R. (2007). Clinical importance and therapeutic implications of the pivotal CXCL12-CXCR4 (chemokine ligand-receptor) interaction in cancer cell migration. *Tumour Biol.* 28 123–131. 10.1159/000102979 17510563

[B5] Ashour A. E. Abd-Allah A. R. Korashy H. M. Attia S. M. Alzahrani A. Z. Saquib Q. (2014). Thymoquinone suppression of the human hepatocellular carcinoma cell growth involves inhibition of IL-8 expression, elevated levels of TRAIL receptors, oxidative stress and apoptosis. *Mol. Cell. Biochem.* 389 85–98. 10.1007/s11010-013-1930-1 24399465

[B6] Ashour A. E. Ahmed A. F. Kumar A. Zoheir K. M. Aboul-Soud M. A. Ahmad S. F. (2016). Thymoquinone inhibits growth of human medulloblastoma cells by inducing oxidative stress and caspase-dependent apoptosis while suppressing NF-kappaB signaling and IL-8 expression. *Mol. Cell. Biochem.* 416 141–155. 10.1007/s11010-016-2703-4 27084536

[B7] Bachelder R. E. Wendt M. A. Mercurio A. M. (2002). Vascular endothelial growth factor promotes breast carcinoma invasion in an autocrine manner by regulating the chemokine receptor CXCR4. *Cancer Res.* 15 7203–7206. 12499259

[B8] Balkwill F. (2004a). Cancer and the chemokine network. *Nat. Rev. Cancer* 4 540–550. 10.1038/nrc1388 15229479

[B9] Balkwill F. (2004b). The significance of cancer cell expression of the chemokine receptor CXCR4. *Semin. Cancer Biol.* 14 171–179. 15246052 10.1016/j.semcancer.2003.10.003

[B10] Benovic J. L. Marchese A. (2004). A new key in breast cancer metastasis. *Cancer Cell* 6 429–430. 10.1016/j.ccr.2004.10.017 15542424

[B11] Bhandari D. Trejo J. Benovic J. L. Marchese A. (2007). Arrestin-2 interacts with the ubiquitin-protein isopeptide ligase atrophin-interacting protein 4 and mediates endosomal sorting of the chemokine receptor CXCR4. *J. Biol. Chem.* 282 36971–36979. 10.1074/jbc.M705085200 17947233

[B12] Bishayee A. Sethi G. (2016). Bioactive natural products in cancer prevention and therapy: progress and promise. *Semin. Cancer Biol.* 40–41, 1–3. 10.1016/j.semcancer.2016.08.006 27565447

[B13] Borrello M. G. Alberti L. Fischer A. Degl’innocenti D. Ferrario C. Gariboldi M. (2005). Induction of a proinflammatory program in normal human thyrocytes by the RET/PTC1 oncogene. *Proc. Natl. Acad. Sci. U.S.A.* 102 14825–14830. 10.1073/pnas.0503039102 16203990 PMC1253545

[B14] Bray F. Jemal A. Grey N. Ferlay J. Forman D. (2012). Global cancer transitions according to the human development index (2008-2030): a population-based study. *Lancet Oncol.* 13 790–801. 10.1016/S1470-2045(12)70211-5 22658655

[B15] Busch C. Krochmann J. Drews U. (2013). The chick embryo as an experimental system for melanoma cell invasion. *PLoS One* 8:e53970. 10.1371/journal.pone.0053970 23342051 PMC3544663

[B16] Chua A. W. Hay H. S. Rajendran P. Shanmugam M. K. Li F. Bist P. (2010). Butein downregulates chemokine receptor CXCR4 expression and function through suppression of NF-kappaB activation in breast and pancreatic tumor cells. *Biochem. Pharmacol.* 80 1553–1562. 10.1016/j.bcp.2010.07.045 20699088

[B17] Contag C. H. Jenkins D. Contag P. R. Negrin R. S. (2000). Use of reporter genes for optical measurements of neoplastic disease *in vivo*. *Neoplasia* 2 41–52. 10.1038/sj.neo.7900079 10933067 PMC1550286

[B18] Dai X. Ahn K. S. Kim C. Siveen K. S. Ong T. H. Shanmugam M. K. (2015). Ascochlorin, an isoprenoid antibiotic inhibits growth and invasion of hepatocellular carcinoma by targeting STAT3 signaling cascade through the induction of PIAS3. *Mol. Oncol.* 9 818–833. 10.1016/j.molonc.2014.12.008 25624051 PMC5528777

[B19] Dey A. Wong E. Kua N. Teo H. L. Tergaonkar V. Lane D. (2008). Hexamethylene bisacetamide (HMBA) simultaneously targets AKT and MAPK pathway and represses NF kappaB activity: implications for cancer therapy. *Cell Cycle.* 7 3759–3767. 10.4161/cc.7.23.7213 19029824

[B20] El Mezayen R. El Gazzar M. Nicolls M. R. Marecki J. C. Dreskin S. C. Nomiyama H. (2006). Effect of thymoquinone on cyclooxygenase expression and prostaglandin production in a mouse model of allergic airway inflammation. *Immunol. Lett.* 106 72–81. 10.1016/j.imlet.2006.04.012 16762422

[B21] El-Mahdy M. A. Zhu Q. Wang Q. E. Wani G. Wani A. A. (2005). Thymoquinone induces apoptosis through activation of caspase-8 and mitochondrial events in p53-null myeloblastic leukemia HL-60 cells. *Int. J. Cancer* 117 409–417. 10.1002/ijc.21205 15906362

[B22] El-Mahmoudy A. Matsuyama H. Borgan M. A. Shimizu Y. El-Sayed M. G. Minamoto N. (2002). Thymoquinone suppresses expression of inducible nitric oxide synthase in rat macrophages. *Int. Immunopharmacol.* 2 1603–1611. 10.1016/S1567-5769(02)00139-X 12433061

[B23] Fernandis A. Z. Prasad A. Band H. Klosel R. Ganju R. K. (2004). Regulation of CXCR4-mediated chemotaxis and chemoinvasion of breast cancer cells. *Oncogene* 23 157–167. 10.1038/sj.onc.1206910 14712221

[B24] Gali-Muhtasib H. Diab-Assaf M. Boltze C. Al-Hmaira J. Hartig R. Roessner A. (2004). Thymoquinone extracted from black seed triggers apoptotic cell death in human colorectal cancer cells via a p53-dependent mechanism. *Int. J. Oncol.* 25 857–866. 15375533

[B25] Gali-Muhtasib H. Roessner A. Schneider-Stock R. (2006). Thymoquinone: a promising anti-cancer drug from natural sources. *Int. J. Biochem. Cell Biol.* 38 1249–1253. 10.1016/j.biocel.2005.10.009 16314136

[B26] Gatti M. Pattarozzi A. Bajetto A. Wurth R. Daga A. Fiaschi P. (2013). Inhibition of CXCL12/CXCR4 autocrine/paracrine loop reduces viability of human glioblastoma stem-like cells affecting self-renewal activity. *Toxicology* 314 209–220. 10.1016/j.tox.2013.10.003 24157575

[B27] Hasanpourghadi M. Looi C. Y. Pandurangan A. K. Sethi G. Wong W. F. Mustafa M. R. (2017). Phytometabolites targeting the warburg effect in cancer cells: a mechanistic review. *Curr. Drug Targets* 18 1086–1094. 10.2174/1389450117666160401124842 27033190

[B28] Helbig G. Christopherson K. W. Bhat-Nakshatri P. Kumar S. Kishimoto H. Miller K. D. (2003). NF-kappaB promotes breast cancer cell migration and metastasis by inducing the expression of the chemokine receptor CXCR4. *J. Biol. Chem.* 278 21631–21638. 10.1074/jbc.M300609200 12690099

[B29] Hung C. S. Su H. Y. Liang H. H. Lai C. W. Chang Y. C. Ho Y. S. (2014). High-level expression of CXCR4 in breast cancer is associated with early distant and bone metastases. *Tumour Biol.* 35 1581–1588. 10.1007/s13277-013-1218-9 24101191

[B30] Hwang Y. S. Han S. S. Kim K. R. Ye-Jin L. Sun-Kyung L. Kwang-Kyun P. (2015). Validating of the pre-clinical mouse model for metastatic breast cancer to the mandible. *J. Appl. Oral. Sci.* 23 3–8. 10.1590/1678-775720140158 25760261 PMC4349112

[B31] Jenkins D. E. Oei Y. Hornig Y. S. Yu S. F. Dusich J. Purchio T. (2003a). Bioluminescent imaging (BLI) to improve and refine traditional murine models of tumor growth and metastasis. *Clin. Exp. Metastasis* 20 733–744. 14713107 10.1023/b:clin.0000006815.49932.98

[B32] Jenkins D. E. Yu S. F. Hornig Y. S. Purchio T. Contag P. R. (2003b). In vivo monitoring of tumor relapse and metastasis using bioluminescent PC-3M-luc-C6 cells in murine models of human prostate cancer. *Clin. Exp. Metastasis* 20 745–756. 14713108 10.1023/b:clin.0000006817.25962.87

[B33] Jia L. Y. Shanmugam M. K. Sethi G. Bishayee A. (2016). Potential role of targeted therapies in the treatment of triple-negative breast cancer. *Anticancer Drugs* 27 147–155. 10.1097/CAD.0000000000000328 26682525

[B34] Kaseb A. O. Chinnakannu K. Chen D. Sivanandam A. Tejwani S. Menon M. (2007). Androgen receptor and E2F-1 targeted thymoquinone therapy for hormone-refractory prostate cancer. *Cancer Res.* 67 7782–7788. 10.1158/0008-5472.CAN-07-1483 17699783

[B35] Kato M. Kitayama J. Kazama S. Nagawa H. (2003). Expression pattern of CXC chemokine receptor-4 is correlated with lymph node metastasis in human invasive ductal carcinoma. *Breast Cancer Res.* 5 R144–R150. 10.1186/bcr627 12927045 PMC314431

[B36] Keeley E. C. Mehrad B. Strieter R. M. (2010). CXC chemokines in cancer angiogenesis and metastases. *Adv. Cancer Res.* 106 91–111. 10.1016/S0065-230X(10)06003-3 20399957 PMC3069502

[B37] Kukreja P. Abdel-Mageed A. B. Mondal D. Liu K. Agrawal K. C. (2005). Up-regulation of CXCR4 expression in PC-3 cells by stromal-derived factor-1alpha (CXCL12) increases endothelial adhesion and transendothelial migration: role of MEK/ERK signaling pathway-dependent NF-kappaB activation. *Cancer Res.* 65 9891–9898. 10.1158/0008-5472.CAN-05-1293 16267013

[B38] Kulbe H. Hagemann T. Szlosarek P. W. Balkwill F. R. Wilson J. L. (2005). The inflammatory cytokine tumor necrosis factor-alpha regulates chemokine receptor expression on ovarian cancer cells. *Cancer Res.* 65 10355–10362. 10.1158/0008-5472.CAN-05-0957 16288025

[B39] Kumar G. Manjunatha B. (2013). Metastatic tumors to the jaws and oral cavity. *J. Oral. Maxillofac Pathol.* 17 71–75. 10.4103/0973-029X.110737 23798834 PMC3687193

[B40] Lee J. H. Kim B. Jin W. J. Kim J. W. Kim H. H. Ha H. (2014). Trolox inhibits osteolytic bone metastasis of breast cancer through both PGE2-dependent and independent mechanisms. *Biochem. Pharmacol.* 91 51–60. 10.1016/j.bcp.2014.06.005 24929117

[B41] Li F. Rajendran P. Sethi G. (2010). Thymoquinone inhibits proliferation, induces apoptosis and chemosensitizes human multiple myeloma cells through suppression of signal transducer and activator of transcription 3 activation pathway. *Br. J. Pharmacol.* 161 541–554. 10.1111/j.1476-5381.2010.00874.x 20880395 PMC2990154

[B42] Li F. Zhang J. Arfuso F. Chinnathambi A. Zayed M. E. Alharbi S. A. (2015). NF-kappaB in cancer therapy. *Arch. Toxicol.* 89 711–731. 10.1007/s00204-015-1470-4 25690730

[B43] Libura J. Drukala J. Majka M. Tomescu O. Navenot J. M. Kucia M. (2002). CXCR4-SDF-1 signaling is active in rhabdomyosarcoma cells and regulates locomotion, chemotaxis, and adhesion. *Blood* 100 2597–2606. 10.1182/blood-2002-01-0031 12239174

[B44] Liu L. Ahn K. S. Shanmugam M. K. Wang H. Shen H. Arfuso F. (2018). Oleuropein induces apoptosis via abrogating NF-kappaB activation cascade in estrogen receptor-negative breast cancer cells. *J. Cell. Biochem.* 10.1002/jcb.27738 [Epub ahead of print]. 30260018

[B45] Manu K. A. Shanmugam M. K. Ong T. H. Subramaniam A. Siveen K. S. Perumal E. (2013). Emodin suppresses migration and invasion through the modulation of CXCR4 expression in an orthotopic model of human hepatocellular carcinoma. *PLoS One* 8:e57015. 10.1371/journal.pone.0057015 23472074 PMC3589458

[B46] Manu K. A. Shanmugam M. K. Rajendran P. Li F. Ramachandran L. Hay H. S. (2011). Plumbagin inhibits invasion and migration of breast and gastric cancer cells by downregulating the expression of chemokine receptor CXCR4. *Mol. Cancer* 10:107. 10.1186/1476-4598-10-107 21880153 PMC3175200

[B47] Muller A. Homey B. Soto H. Ge N. Catron D. Buchanan M. E. (2001). Involvement of chemokine receptors in breast cancer metastasis. *Nature* 410 50–56. 10.1038/35065016 11242036

[B48] Mundy G. R. (2002). Metastasis to bone: causes, consequences and therapeutic opportunities. *Nat. Rev. Cancer* 2 584–593. 10.1038/nrc867 12154351

[B49] Perez E. A. Spano J. P. (2012). Current and emerging targeted therapies for metastatic breast cancer. *Cancer* 118 3014–3025. 10.1002/cncr.26356 22006669

[B50] Puar Y. R. Shanmugam M. K. Fan L. Arfuso F. Sethi G. Tergaonkar V. (2018). Evidence for the involvement of the master transcription factor NF-kappaB in cancer initiation and progression. *Biomedicines* 27:6. 10.3390/biomedicines6030082 30060453 PMC6163404

[B51] Rehemtulla A. Stegman L. D. Cardozo S. J. Gupta S. Hall D. E. Contag C. H. (2000). Rapid and quantitative assessment of cancer treatment response using *in vivo* bioluminescence imaging. *Neoplasia* 2 491–495. 10.1038/sj.neo.7900121 11228541 PMC1508085

[B52] Roepke M. Diestel A. Bajbouj K. Walluscheck D. Schonfeld P. Roessner A. (2007). Lack of p53 augments thymoquinone-induced apoptosis and caspase activation in human osteosarcoma cells. *Cancer Biol. Ther.* 6 160–169. 10.4161/cbt.6.2.3575 17218778

[B53] Salem M. L. (2005). Immunomodulatory and therapeutic properties of the *Nigella sativa* L. seed. *Int. Immunopharmacol.* 5 1749–1770. 10.1016/j.intimp.2005.06.008 16275613

[B54] Scatena C. D. Hepner M. A. Oei Y. A. Dusich J. M. Yu S. F. Purchio T. (2004). Imaging of bioluminescent LNCaP-luc-M6 tumors: a new animal model for the study of metastatic human prostate cancer. *Prostate* 59 292–303. 10.1002/pros.20003 15042605

[B55] Schioppa T. Uranchimeg B. Saccani A. Biswas S. K. Doni A. Rapisarda A. (2003). Regulation of the chemokine receptor CXCR4 by hypoxia. *J. Exp. Med.* 198 1391–1402. 10.1084/jem.20030267 14597738 PMC2194248

[B56] Sethi G. Ahn K. S. Aggarwal B. B. (2008a). Targeting nuclear factor-kappa B activation pathway by thymoquinone: role in suppression of antiapoptotic gene products and enhancement of apoptosis. *Mol. Cancer Res.* 6 1059–1070. 10.1158/1541-7786.MCR-07-2088 18567808

[B57] Sethi G. Shanmugam M. K. Ramachandran L. Kumar A. P. Tergaonkar V. (2012). Multifaceted link between cancer and inflammation. *Biosci. Rep.* 32 1–15. 10.1042/BSR20100136 21981137

[B58] Sethi G. Sung B. Aggarwal B. B. (2008b). Nuclear factor-kappaB activation: from bench to bedside. *Exp. Biol. Med.* 233 21–31. 10.3181/0707-MR-196 18156302

[B59] Sethi G. Tergaonkar V. (2009). Potential pharmacological control of the NF-kappaB pathway. *Trends Pharmacol. Sci.* 30 313–321. 10.1016/j.tips.2009.03.004 19446347

[B60] Shanmugam M. K. Arfuso F. Kumar A. P. Wang L. Goh B. C. Ahn K. S. (2018). Modulation of diverse oncogenic transcription factors by thymoquinone, an essential oil compound isolated from the seeds of *Nigella sativa* Linn. *Pharmacol. Res.* 129 357–364. 10.1016/j.phrs.2017.11.023 29162539

[B61] Shanmugam M. K. Kannaiyan R. Sethi G. (2011a). Targeting cell signaling and apoptotic pathways by dietary agents: role in the prevention and treatment of cancer. *Nutr. Cancer* 63 161–173. 10.1080/01635581.2011.523502 21294053

[B62] Shanmugam M. K. Manu K. A. Ong T. H. Ramachandran L. Surana R. Bist P. (2011b). Inhibition of CXCR4/CXCL12 signaling axis by ursolic acid leads to suppression of metastasis in transgenic adenocarcinoma of mouse prostate model. *Int. J. Cancer* 129 1552–1563. 10.1002/ijc.26120 21480220

[B63] Shanmugam M. K. Rajendran P. Li F. Nema T. Vali S. Abbasi T. (2011c). Ursolic acid inhibits multiple cell survival pathways leading to suppression of growth of prostate cancer xenograft in nude mice. *J. Mol. Med.* 89 713–727. 10.1007/s00109-011-0746-2 21465181

[B64] Shanmugam M. K. Kumar A. P. Tan B. K. H. Sethi G. (2013). Role of NF-κB in tumorigenesis. *For. Immunopathol. Dis. Ther.* 4 187–203. 10.1615/ForumImmunDisTher.2013008382

[B65] Shanmugam M. K. Lee J. H. Chai E. Z. Kanchi M. M. Kar S. Arfuso F. (2016). Cancer prevention and therapy through the modulation of transcription factors by bioactive natural compounds. *Semin. Cancer Biol.* 40–41 35–47. 10.1016/j.semcancer.2016.03.005 27038646

[B66] Shanmugam M. K. Nguyen A. H. Kumar A. P. Tan B. K. Sethi G. (2012). Targeted inhibition of tumor proliferation, survival, and metastasis by pentacyclic triterpenoids: potential role in prevention and therapy of cancer. *Cancer Lett.* 320 158–170. 10.1016/j.canlet.2012.02.037 22406826

[B67] Shanmugam M. K. Rane G. Kanchi M. M. Arfuso F. Chinnathambi A. Zayed M. E. (2015). The multifaceted role of curcumin in cancer prevention and treatment. *Molecules* 20 2728–2769. 10.3390/molecules20022728 25665066 PMC6272781

[B68] Shoieb A. M. Elgayyar M. Dudrick P. S. Bell J. L. Tithof P. K. (2003). In vitro inhibition of growth and induction of apoptosis in cancer cell lines by thymoquinone. *Int. J. Oncol.* 22 107–113. 10.3892/ijo.22.1.107 12469192

[B69] Siveen K. S. Ahn K. S. Ong T. H. Shanmugam M. K. Li F. Yap W. N. (2014a). Y-tocotrienol inhibits angiogenesis-dependent growth of human hepatocellular carcinoma through abrogation of AKT/mTOR pathway in an orthotopic mouse model. *Oncotarget* 5 1897–1911. 24722367 10.18632/oncotarget.1876 PMC4039111

[B70] Siveen K. S. Mustafa N. Li F. Kannaiyan R. Ahn K. S. Kumar A. P. (2014b). Thymoquinone overcomes chemoresistance and enhances the anticancer effects of bortezomib through abrogation of NF-kappaB regulated gene products in multiple myeloma xenograft mouse model. *Oncotarget* 5 634–648. 24504138 10.18632/oncotarget.1596 PMC3996662

[B71] Staller P. Sulitkova J. Lisztwan J. Moch H. Oakeley E. J. Krek W. (2003). Chemokine receptor CXCR4 downregulated by von hippel-lindau tumour suppressor pVHL. *Nature* 425 307–311. 10.1038/nature01874 13679920

[B72] Sys G. M. Lapeire L. Stevens N. Favoreel H. Forsyth R. Bracke M. (2013). The in ovo CAM-assay as a xenograft model for sarcoma. *J. Vis. Exp.* 17:e50522. 10.3791/50522 23892612 PMC3845689

[B73] Tang C. H. Sethi G. Kuo P. L. (2014). Novel medicines and strategies in cancer treatment and prevention. *Biomed. Res. Int.* 2014:474078. 10.1155/2014/474078 24971330 PMC4058237

[B74] Torre L. A. Bray F. Siegel R. L. Ferlay J. Lortet-Tieulent J. Jemal A. (2015). Global cancer statistics, 2012. *CA Cancer J. Clin.* 65 87–108. 10.3322/caac.21262 25651787

[B75] van der Horst G. van der Pluijm G. (2012). Preclinical models that illuminate the bone metastasis cascade. *Recent Results Cancer Res.* 192 1–31. 10.1007/978-3-642-21892-7_1 22307368

[B76] Wang C. Kar S. Lai X. Cai W. Arfuso F. Sethi G. (2018). Triple negative breast cancer in Asia: an insider’s view. *Cancer Treat. Rev.* 62 29–38. 10.1016/j.ctrv.2017.10.014 29154023

[B77] Woo C. C. Hsu A. Kumar A. P. Sethi G. Tan K. H. (2013). Thymoquinone inhibits tumor growth and induces apoptosis in a breast cancer xenograft mouse model: the role of p38 MAPK and ROS. *PLoS One* 8:e75356. 10.1371/journal.pone.0075356 24098377 PMC3788809

[B78] Woo C. C. Kumar A. P. Sethi G. Tan K. H. (2012). Thymoquinone: potential cure for inflammatory disorders and cancer. *Biochem. Pharmacol.* 83 443–451. 10.1016/j.bcp.2011.09.029 22005518

[B79] Woo C. C. Loo S. Y. Gee V. Yap C. W. Sethi G. Kumar A. P. (2011). Anticancer activity of thymoquinone in breast cancer cells: possible involvement of PPAR-gamma pathway. *Biochem. Pharmacol.* 82 464–475. 10.1016/j.bcp.2011.05.030 21679698

[B80] Worthen D. R. Ghosheh O. A. Crooks P. A. (1998). The in vitro anti-tumor activity of some crude and purified components of blackseed, *Nigella sativa* L. *Anticancer Res.* 18 1527–1532. 9673365

[B81] Xu C. Zhao H. Chen H. Yao Q. (2015). CXCR4 in breast cancer: oncogenic role and therapeutic targeting. *Drug Des. Dev. Ther.* 9 4953–4964. 10.2147/DDDT.S84932 26356032 PMC4560524

[B82] Yang S. F. Weng C. J. Sethi G. Hu D. N. (2013). Natural bioactives and phytochemicals serve in cancer treatment and prevention. *Evid. Based Comp. Alternat. Med.* 2013:698190. 10.1155/2013/698190 24454507 PMC3880702

[B83] Yi T. Cho S. G. Yi Z. Pang X. Rodriguez M. Wang Y. (2008). Thymoquinone inhibits tumor angiogenesis and tumor growth through suppressing AKT and extracellular signal-regulated kinase signaling pathways. *Mol. Cancer Ther.* 7 1789–1796. 10.1158/1535-7163.MCT-08-0124 18644991 PMC2587125

[B84] Zhang J. Ahn K. S. Kim C. Shanmugam M. K. Siveen K. S. Arfuso F. (2016). Nimbolide-induced oxidative stress abrogates STAT3 signaling cascade and inhibits tumor growth in transgenic adenocarcinoma of mouse prostate model. *Antioxid. Redox Signal.* 24 575–589. 10.1089/ars.2015.6418 26649526

[B85] Zlotnik A. (2006). Chemokines and cancer. *Int. J. Cancer* 119 2026–2029. 10.1002/ijc.22024 16671092

